# War of words: interculturalism v. multiculturalism

**DOI:** 10.1186/s40878-018-0079-1

**Published:** 2018-05-17

**Authors:** Christian Joppke

**Affiliations:** Institute of Sociology, University of Bern, Fabrikstrasse 8, CH-3012 Bern, Switzerland

## Abstract

This article tackles the relationship between interculturalism and multiculturalism from the points of view of both. Interculturalism owes its existence to a critique of multiculturalism, but of highly distorted visions of it. I distinguish between two versions of interculturalism, a majoritarian (practiced in Québec) and a post-majoritarian (in Europe), which yield diametrically opposed visions of multiculturalism, as either footloose cosmopolitan or parochial-segregationist. Among the problems of interculturalism is the vacuity of the local as its preferred site of intervention, and its rushed embracing of “diversity” that is also a central plank of neoliberal ideology.

## Introduction

Over the past decade or so, "interculturalism" has established itself as new approach to manage cultural diversity in liberal-democratic societies (a comprehensive overview is Grillo, 2017). While its near-namesake "multiculturalism" is heavily contested or even discarded in more and more places, especially in Europe, interculturalism enjoys unbridled support, particularly among policy elites, and it has been firmly institutionalized, for instance, in pedagogy or social work, whose curricula now include required courses in "intercultural competence". In Germany, never a standard-bearer of multiculturalism, recent integration laws passed in several Länder prominently sport the "intercultural opening of public administration", which requires the state bureaucracy to be attentive to the linguistic, religious, and other cultural needs of its immigrant-based clientele. The latter even entails prioritizing the hiring of people with a "migration background" (*mit Migrationshintergrund*), now the official speak for the near 20% of the German resident population with at least one foreign parent.

Several elements mark interculturalism:

- a stress on communication and "dialogue" across ethnic (as still the most critical of cultural) boundaries—hence the "inter" in the name;
- a preference for the local over the national as site of policy intervention and group and interpersonal exchange;
- a concrete policy as against abstract theoretical focus, and closely related, a preference for pragmatic and compromise-minded over principled reasoning and decision-making;
- finally, a non-ideological diction and cross-party appeal (all these elements can be found in Zapata-Barrero, 2017).

In each of these respects interculturalism derives its distinction in contrast to a discarded multiculturalism,

- as favoring group separation over inter-group exchange and finding a common ground;
- as being into national-level grand posturing while ignoring the local *hic et nunc*;
- as being largely theoretical and aloof of concrete policy concerns;
- and as showing itself unprepared for compromising its radical and purist agenda.

One understands that particularly moderate and liberal defenders of multiculturalism (others are hardly around anymore) are not amused, rejecting the intercultural critique as “a misrepresentation, even caricature, of multiculturalism theories and approaches” (Kymlicka, 2016, p. 158) (see also Modood, 2017, p. 2).

## Mutual (mis) perceptions

A closer look reveals the intercultural critique of multiculturalism and the multicultural meta-critique, as well as the suggested affinities and oppositions from both parties’ point of view, to be more variegated and less categorical than first meets the eye. Let us look at two prominent examples on each side, beginning with the interculturalists. Perhaps the most strident of them is Cantle, a British community organizer who became famous for launching the British policy turn, after a wave of race riots in several northern English towns in the summer of 2001, from multiculturalism to ‘community cohesion’. He sees little in common with an opposed multiculturalism, promoting interculturalism as “a completely different concept which reflects the new realities of diversity” (Cantle, 2016, p. 472). Apart from faulting multiculturalism for having fueled “parallel lives” and group segregation (which is interculturalism’s standard charge that has never been backed by solid evidence), he finds it guilty of having prioritized “race (and class)” (a very British combination, alluding to the radical journal under that name) over other forms of diversity, many of which are more on the voluntary than strictly ascribed pole, like “mixed race”, “sexual orientation”, or “faith differences”, and which he sees growing in the contemporary period. He cites the example of a “Glaswegian, Pakistani teenager of Muslim descent who supports Glasgow Rangers in a Catholic School” (Cantle, 2016, p. 476), who is not well served by a “tick-box classification which homogenizes communities under the single aspect of their identities” (Cantle, 2016, p. 477). However, even if this youngster may hold “a different identity at home compared to that at school”, Cantle’s assumption that “she might have a different identity next week” (Cantle, 2016, p. 477) may go a bit far. Modood (2016, p. 487) rightly retorts that some identities are more painfully experienced and disadvantaging than others, and that it is *these* that are targeted by multiculturalism: “We cannot require all minorities to wear their identities lightly, flexibly and contextually.” One does not have to endorse Modood’s “groupist” multiculturalism to question interculturalism’s extreme multiplication of identities, which also militates against the policy relevance that interculturalists otherwise pride themselves for.

A less combative note is struck by a second prominent defender of interculturalism, Zapata-Barrero (2017), who depicts interculturalism not as substitutive of but “complementary” to multiculturalism. He even argues that a fully developed “recognition of rights” (which he attributes to multiculturalism) is necessary for interculturalism’s “contact”-orientation to take off (p. 6). In Zapata-Barrero’s view, interculturalism arises in a “post-multicultural” moment of anti-multiculturalist backlash, financial crisis (favoring low-cost, “mainstreaming” policy solutions to diversity), and “superdiversity” with its multiple identity and post-race issues, where diversity is even eroding the notion of a homogeneous “majority”. More concretely, interculturalism figures for him as “mediator” between, on the one side, an increasingly discarded multiculturalism and, on the other side, duty-focused civic integration policies, whose impulse of limits to tolerance and of finding a common ground is taken on board by his (and all other versions of) interculturalism. For Zapata-Barrero, the joint problem of both alternatives, multiculturalism and civic integration, which he sees locked in a vicious circle, is to locate diversity only on the part of minorities, whereby diversity becomes “the other” of the majority, while the need of the day is “placing diversity *within* the mainstream” (p. 6) (this is also the meaning of Cantle’s plea to “live *in* diversity” (Cantle, 2016)). While this sounds plausible as a sociological fact, the implications for policy are again a lot less clear, or rather: frustratingly broad and fungible. Because, if diversity is everywhere it is also nowhere in particular, becoming an empty slogan deployable in almost any direction one likes, by business, for instance (see below).

In the multiculturalist meta-critique, interculturalism is at best “a version of multiculturalism rather than…an alternative paradigm” (Modood, 2017, p. 1). For Modood, interculturalism helps multiculturalism to correct some of its weaknesses, making it more true to itself in the process, as non-essentialist and dialogue-oriented as its presumed opponent and itself a center-building rather than center-fleeing “multicultural nationalism”. In sum, interculturalism and multiculturalism are “critical friends”, not “alternatives” (Modood, 2016, p. 487). Less ground is conceded by Kymlicka (2016), for whom interculturalism serves merely “rhetorical functions”, namely, to continue multiculturalism`s progressive agenda under the mantle of repudiating it in name. But as the price for it is sacking multiculturalism, this risks legitimizing the nationalist populism that has given a bad name to multiculturalism in the first.

This leaves us with a contradictory medley of multiculturalism-interculturalism linkages, from mutually exclusive and substitutive to closely linked if not near-identical.

## Not one but two: majoritarian v. post-majoritarian interculturalism

Much as there is no agreement about what multiculturalism is, it should not surprise that interculturalism, as it has come into existence only by way of a critique of multiculturalism (see Grillo, 2017, chapter 2), shares the problem of its criticized other to be notoriously elusive and difficult to define. But if there are several variants of multiculturalism (see Joppke, 2017, chapter 2), on the part of interculturalism it comes down to just two, which still rest on (or yield) diametrically opposed visions of what multiculturalism is. Let’s call one “majoritarian” interculturalism, which entails the curious vision of multiculturalism as footloose, cosmopolitan diversity, and let’s call the other “post-majoritarian”, for which multiculturalism, on the opposite, is caught in an anachronistic majority-minority dualism.

Majoritarian interculturalism has its roots in francophone Québec and the province’s nationalist-cum-secessionist leanings within Anglophone Canada. Interculturalism is Québec’s answer to Canadian multiculturalism, which it has always rejected for watering-down its nationalist ambition. In Bouchard’s (2011) formulation, interculturalism is a code word for “majority precedence”, that is, the respect for and acceptance of the francophone majority culture by immigrants. This includes acceptance of French as public language (including the controversial obligation on immigrants to have their children schooled in French), “predominance to the majority narrative in national memory and history courses” (“je me souviens” is Québec’s official motto, adorning the province’s car license plates), the privileging of Christianity in the school curriculum, the burial of heads of State in Catholic churches, to keep the Christian cross on the Québec flag, Christmas decoration in public squares and buildings, and the sounding bells of Catholic churches (Bouchard, 2011, p. 459). Bouchard calls this majority precedence “ad hoc” or “contextual”, distinguishing it from the “formal” precedence that, for instance, France imposes on its immigrants in terms of Republicanism. Indeed, what is striking in Bouchard’s catalogue is the predominance of linguistic and religious entries and, conversely, the complete absence of universalist and more political values or principles, such as freedom or equality, which are central to most other country’s pluralism-containing core commitments. This peculiarity is due to the fact that universalist-political values can’t distinguish Québec from the rest of Canada. Quebecois particularism flies because “the francophone majority is itself a precarious minority that needs protection in order to ensure its survival” (Bouchard, 2011, p. 441).

This raises the interesting question whether “majority precedence” as the defining feature of majoritarian interculturalism is limited to the specific situation of Québec - a minority nation within a multinational state - or is generalizable to all nation-states in which there is a majority not wishing to be weakened or even extinguished by immigration. We shall see that every form of interculturalism will stress the importance of core values and principles that it does not want to see compromised by immigrant diversity. However, outside Québec this common core is strictly universalist and limited to the exigencies of liberal democracy itself (including a more functional approach to language as medium of communication rather than badge of identity).

What is “multiculturalism” according to an interculturalism that is primarily protective of “majority culture” (Bouchard, 2011, p. 438)? First, it denies any communality between the two (though in effect, there may be little difference between them, as argues Taylor, 2012), and as a matter of fact all Québec governments have rejected Canadian multiculturalism ever since it was introduced in 1971. More controversially, Canadian multiculturalism, and with it all other forms of multiculturalism in Australia, Sweden, the United States, or India, are seen by Bouchard (2011 p. 441) as beholden to a “diversity” paradigm, in which there is “no recognition of a majority culture.” This is as if Patten’s (2014) plea for a neutrality-based multiculturalism of states without a *Favoritvolk* had come true. This may make sense for Canada or Australia, whose historical majority culture was an ancestral Britishness that had strongly racial connotations, excluding through their immigration and citizenship policies all Asians or Africans well into the 1960s, and even longer in Australia. However, Bouchard’s equation of multiculturalism with a symmetric diversity policy that does not recognize a majority culture greatly conflates different nation-state amalgams and resulting multiculturalism forms; above all, it denies the possibility, stressed by some of its theorists from Modood to Kymlicka, that multiculturalism itself could become a defining mark of majority culture if not of nationhood, which seems to be the case precisely for post-racist settler states like Canada and Australia (but not the United States, which separated from Britain early and disposes of a strong own founding myth).

One might think that majoritarian interculturalism, which operates with a majority-minority dualism, would provide a model for Europe, with its ethnic nation-state legacies (this is the assumption by Taylor, 2012). In reality, a different, post-majoritarian form of interculturalism has emerged in Europe. Its central document is the Council of Europe’s White Paper on Intercultural Dialogue, entitled “Living Together as Equals in Dignity” (Council of Europe, 2008). Espousing the interculturalism that is recognizable in the pages of Cantle or Zapata-Barrero, the White Paper advocates “intercultural dialogue” as alternative to a multiculturalism that “had been found inadequate” in this respect, on the one hand, and to an anyway long-denigrated “assimilation”, on the other hand. Both are found guilty of “the same, schematic conception of society set in opposition of majority and minority, differing only in endorsing separation of the minority from the majority…(or) assimilation to it” (Council of Europe, 2008, p. 18). Whereas in Québec-style majoritarian interculturalism “diversity” is lodged on the opposite side of a majority-denying, cosmopolitan multiculturalism, here the starting assumption is the fact of “unprecedented and ever-growing” diversity (p. 9) this side of the divide, rendering anachronistic “old approaches”, multiculturalism included. Obviously, the new approach wanes diversity on *its* side, as argument to go beyond the groupist majority-minority binary that multiculturalism is said to be caught in while fueling “communal segregation” and “mutual incomprehension” (p. 19).

While the “multiculturalism” appearing through the majoritarian interculturalism lens barely matched any real-world multiculturalism, in fact transformed it into cosmopolitanism, post-majoritarian interculturalism suffers from the reverse problem of reduplicating the grossest stereotypes about multiculturalism, as separatist and “undermining the rights of individuals”, women in particular (Council of Europe, 2008, p. 19). Therefore there is the same emphasis on fleshing out a common core as in majoritarian interculturalism, only that in the post-majoritarian variant the common core is not particularist but universalist, consisting of the core principles of liberal democracy. The “common identity”, which multiculturalism is accused of to have slighted or even violated, is dubbed “equality of human dignity”, “an ethos of respect for the equal dignity of every individual and hospitality towards the wider world” (p. 14). Furthermore, “intrinsic to such an ethos is dialogue and interaction with others” (p. 14). Distinguishing itself from multiculturalism and assimilation alike, interculturalism thus understood “incorporates the best of both. It takes from assimilation the focus on the individual; it takes from multiculturalism the recognition of cultural diversity. And it adds the new element, critical to integration and social cohesion, of dialogue and the basis of equal dignity and shared values” (p. 19).

Alas, which multiculturalist would deny the “equality of human dignity”, which in Taylor’s multicultural Programmschrift (Taylor, 1994) is exactly the starting-point for the modern “politics of equal recognition”, of which multiculturalism is only the difference-emphasizing variant? Which multiculturalist, in turn, would advance a total relativism of values and cultures, including honor killings, genital mutilation and other extreme practices, as seems to be the charge of the interculturalists? Moreover, when it comes to concrete policy proposals, the Council of Europe White Paper (2008) offers little beyond the standard fare long known from the liberal multiculturalism agenda: “positive action” (p. 39), “clear legislation and policies against discrimination” (p. 37), and local voting rights for immigrants (p. 42). Moreover, if “every form of stigmatization of persons belonging to minority and disadvantaged groups in public discourse needs to be ruled out” (p. 42), interculturalism even seems to endorse the restrictions on free speech and imposition of behavioural etiquettes that multiculturalists like Tariq Modood and Bhikhu Parekh, controversially (see the debate edited by Hansen, 2006), have demanded during the perennial Islam conflicts, from Rushdie’s Satanic Verses to the Danish Cartoons.

## Multiculturalism in a liberal register

The pros and cons of multiculturalism have been extensively debated, so there is no need to repeat this here. Suffice it to say that, in my view (Joppke, 2017), the legal-constitutional resources of liberal states are more capacious than even the most convincing liberal theories of multiculturalism, those by Kymlicka (1995) and Patten (2014), have realized. So there is less need for explicitly “multicultural” policy commitments than commonly believed. Take again the case of Germany, which is an interesting case for its political hostility to but legal embracing of multiculturalism. In the recent jurisdiction of its higher courts, one can register an interesting equilibrium between multicultural and more centrist, civic commitments, and one could not even say where one ends and the other begins. For instance, for the sake of being socialized into a pluralistic society with many diverse lifestyles, Muslim school girls must stomach the view of boys in “tightly cut swimwear”, so that there is no exemption for them from co-educational swimming lessons in public schools.^1^ However, the same endorsement of pluralism implies, in turn, that non-Muslim pupils must endure the sight of a headscarf-wearing school teacher, who is only exercising the constitutional right to practice her religion.^2^ While both judgments yielded opposite outcomes - the refusal of a religious exemption request and the validation of an Islamic headscarf claim, respectively - they both affirm cultural pluralism, perhaps a preferable term to the spent “multiculturalism”. There is no alternative to pluralism and diversity, protected by the law, in a liberal society. This becomes especially clear when it is challenged, which is no rare event in the current climate of nationalist populism. Take German Interior Minister Thomas de Mazières recent foray for a “German *Leitkultur*”, strangely entitled “We Are Not Burka”.^3^ He cites the example of shaking hands as part of the German way of life. This is an informal practice that, of course, is not prescribed by law, and thus it goes beyond mere “constitutional patriotism” as the minimal integration requirement of a liberal society. Instead, the handshake points to something that keeps together German society “*im Innersten*” (in the innermost), and that “defines us and distinguishes us from others”, hence, the Germans’ *Leitkultur*. Habermas (2017) quickly responded that as informal cultural practice the handshake could not be made a legal obligation—then the “high five”, popular not only among youngsters, would have to be prohibited. Habermas overlooked that the Interior Minster saw that himself: “The word *leiten* (leading) means something else than to prescribe (*vorschreiben*) or to oblige (*verpflichten*)”, one can read in his pamphlet. His plea for a German *Leitkultur* does not entail the renunciation of pluralism. Multiculturalism is not dead but very much alive in Germany, despite Chancellor Merkel’s famous diction that it has “utterly failed”.

## Vacuity of the local

Multiculturalism is actually a much better, because nicely ambiguous and multivocal term for the pluralism that is inevitable in a liberal society than the neologism “interculturalism”. The drawback of the latter is to immediately point to a program or policy under its name. But which? From the city context, the true home ground of interculturalism, one knows that the “inter” refers to mayoral support for “projects” rather than “groups” (Uitermark, Rossi, & Van Houtum, 2005). But is there anything more to say than what this or that city does in the name of the “inter”, which might just as well go under the label “multi”, or under no particular label at all, as in “keeping things together”, which is Amsterdam charismatic mayor Job Cohen`s description of his pragmatic approach? In Zapata-Barrero’s (2017, p. 141) appraisal of local and policy-savvy interculturalism, we learn that the main “concern” is “over how to manage contact in public spaces”. He lists the following “spaces” where “contact” is to occur: “community gardens, libraries, public amenities, festivals and neighbourhood spaces” (ibid.). What should one make of this? Communication in such settings is the kind of thing that city or district administrations have *always* and *by definition* been concerned about, what else are public places for? These places are nothing if not used by people, and what else should the latter do in them but talk or listen or read or gaze, *all* of which are communicative acts of sorts, even if the listening is to the whales (inside)? What are the *specifically* “intercultural” elements to this, except that an ethnic line is crossed, which is a trivia because shared humanity must always be at work?

One should mention here that a measure that, of course, is not of the intercultural tool box, the recent burka laws of France, Belgium, or Austria, which prohibit to cover one’s face in all public spaces, are exactly meant to facilitate communication and “dialogue” across ethnic group boundaries. In the French justification of the law before the European Court of Human Rights, the burka interrupts the “reciprocity” and the availability for communication that is said to be the life-blood of “civil society”.^4^ Weirdly, the French government construed it to be the “right of others” that any person, including the pious Muslim woman, be always available for communication at any one time one pleases. As this “right” entails the obligation to always be available for communication, it naturally cannot be found in the European Convention of Human Rights. Of course, no interculturalist endorses forced “contact” of this sort. Because the point about “contact” is that it cannot be forced, at least not in a liberal society worth the name. But then you cannot say more about it than “nice” when it happens (and all goes well in its course), and just register a fact beyond your control, except perhaps trying to establish a “choice architecture” (Thaler & Sunstein, 2008) that may facilitate talk and walk across group lines - say, placing a playground not within but at the intersection of two ethnic neighborhoods. The local as site of integration, though proverbially *the* site of integration, is entirely vacuous for the purposes of theory and better left to those who live and act it out at their discretion - too much state and policy there can only do harm, as in the notorious burka laws.

In fact, the burka laws, except the one in force in the Swiss canton of Ticino, are national laws, which shows the dangers of vacating the national in favor of the local. Kymlicka (2016) pointed out that “local projects of intercultural interaction (will) always be fragile in the absence of an explicit state commitment to redefining nationhood” (p. 172). His multiculturalism theory rests on the assumption that the “link between nationhood and liberal democracy creates systematic risks” for minorities (p. 168), which for the sake of liberal justice have to be compensated by multicultural minority rights. While the prospects for “multicultural nationhood”, which Kymlicka endorses much like Modood, may be somewhat hopeful for most European countries, the impulse not to leave “the nation” to the “exclusionary narrative” of the populists is a valid one (p. 174).

## Problems with diversity

Interculturalists outside Québec have too lightly dismissed the majority-minority binary as anachronistic, and they have gone too far in indigenizing diversity, that is, generalizing it beyond the case of minorities and deeming the very majority category obsolete. It is not yet the case that “most so-called citizens have an immigrant background” (Zapata-Barrero, 2017, p. 15). This does not mean that the majority category is unproblematic, quite the contrary. While interculturalists (outside Québec) dismiss the category as such, even liberals have started to debate whether majorities might need protection today. In democratic theory, majorities have always been viewed with skeptical eyes, as bent on “tyranny” if not checked by liberal constitutions that protect minorities qua individual rights. As Liav Orgad argues sharply, the “inevitable outcome of multiculturalism”, understood as scheme of minority rights, is the concept of a “distinctive cultural majority” (Orgad, 2016, p. 3), which nevertheless remains legally uncharted. Considering recent demographic and immigration trends, with shrinking native populations and growing immigrant diasporas of ever more distant origins, respectively, perhaps the moment has come for something as paradoxical as a “liberal theory of majority rights” (Orgad, 2015). In a way, Orgad generalizes the Quebecois constellation of an embattled minority nation, while wisely endorsing only the European solution of “national constitutionalism” for defining the not-to-be-compromised common core. “National constitutionalism” strongly resembles “constitutional patriotism” as liberal formula of identity and unity in a diverse society. Calling this a theory of *majority* rights may thus be a misnomer, because all cultural particularism, except the one that supports and frames a particular instance of a liberal constitution, has been purged from it. The message is that whatever a “majority” is, it is a site of intense contestation, and interculturalism has too quickly dispensed with it in toto.

Conversely, interculturalists have too quickly embarked on the treacherous train of diversity, including its instrumentalist and business-minded variant of “diversity advantage” (Zapata-Barrero, 2017, p. 13). Kymlicka (2015, p. 21) is more on the mark when attacking “neoliberal multiculturalism” as “inclusion without solidarity”, whereby “equality” is reduced to “equal access to (or perhaps better: equal exposure to) the competitive global marketplace”. When “diversity” was created as a legal term, it was an argument against a remedial-justice oriented interpretation of American civil rights law, and it couched American society in the history-distorting colors of a “nation of minorities”, whites included (see Joppke, 2017, pp. 57–58). From US Supreme Court Justice Powell’s invention on, “diversity” has been devoid of a justice dimension and presented as in the interest of domain-specific efficiency. No wonder that it quickly became a dominant management philosophy, led by the diction that “a more diverse workforce will…bring greater access to new segments of the marketplace” (Kelly & Dobbin, 1998, p. 962). In the United States, diversity management replaced affirmative action programs, which became inopportune during the 1980s Reagan administration and henceforth were associated with ethnic one-upmanship. A manager in Kodak company’s Global Diversity Office puts it thus: “Diversity is not about advancing the agenda of special groups. Diversity includes everyone, and it requires the buy-in and commitment of all of our employees. It is diversity of ideas - not just race” (quoted in Joppke, 2017, p. 59). An add by corporate investment giant Merrill Lynch performs diversity’s reductio ad absurdum: “*Be yourself*. Race. Ethnicity. Religion. Nationality. Gender. Sexual orientation. In the end, there’s just one variety of human being. The individual. All six billion of us. Be bullish. *Merrill Lynch*” (Joppke, 2017, p. 57). If interculturalism buys into “diversity advantage”, as Zapata-Barrero (2017) suggests it does and should, it is cow-towing to neoliberalism.

## War of words

The *querelle* between interculturalism and multiculturalism, picked by an interculturalism that has come into life only as a critique of multiculturalism, is in the first a war of words, despite some different accents here or there set by the interculturalists - in my view, not enough to warrant the neologism. I confer with Kymlicka and Modood’s view that interculturalists give a distorted picture of multiculturalism (particularly its liberal variant), and Kymlicka is also to the point about the mainly “rhetorical functions” and attending risks of the new speak. The great advantage of the multiculturalists is to have, at least, theories to bite one’s teeth into, while the interculturalists have little to nothing on offer in this respect. This does not mean to endorse the multicultural agenda of an explicitly group-recognizing and -protecting policy, which underrates the power of liberal constitutionalism to perform much of its tasks, short of its name. If one of the two terms is to be retained, it should be “multiculturalism”, because of its stubborn justice instincts. “Interculturalism” (outside Québec) concedes too much to the dual reign of neoliberalism and nationalist populism in our globalizing business civilization.

## Footnotes

1 BVerG 6 C 25.12, 11 September 2013.

2 BvR 471/10 and 1 BvR 1181/10, 27 January 2015.

3 First published in the popular tabloid *Bild-Zeitung*, it is reprinted under the strange title “Wir sind nicht Burka” (We Are Not Burka) in *Zeit Online*, 30 April 2017.

4 ECtHR, *S.A.S. v. France*, 1 July 2014.
